# Leptomeningeal carcinomatosis arising from esophageal cancer: a systematic review of the literature

**DOI:** 10.1093/dote/doag126

**Published:** 2026-11-03

**Authors:** Adam Mylonakis, Dimosthenis Stylios, Diamantis Tsilimigras, Georgios Charalampopoulos, Ioannis Kotsantis, Michail Liontos, Ioannis Georgakopoulos, Dimitrios Schizas

**Affiliations:** First Department of Surgery, National and Kapodistrian University of Athens, Laikon General Hospital, Athens, Greece; First Department of Surgery, National and Kapodistrian University of Athens, Laikon General Hospital, Athens, Greece; Department of Surgery, Division of Surgical Oncology, The Ohio State University, Wexner Medical Center and James Comprehensive Cancer Center, Columbus, OH, USA; Second Department of Radiology, National and Kapodistrian University of Athens, Attikon University Hospital, Athens, Greece; Medical Oncology Unit, Second Propaedeutic Department of Medicine, National and Kapodistrian University of Athens, Attikon University Hospital, Athens, Greece; Department of Clinical Therapeutics, Division of Oncology, National and Kapodistrian University of Athens, Alexandra Hospital, Athens, Greece; Radiation Oncology Unit, First Department of Radiology, National and Kapodistrian University of Athens, Aretaieion Hospital, Athens, Greece; First Department of Surgery, National and Kapodistrian University of Athens, Laikon General Hospital, Athens, Greece

**Keywords:** central nervous system neoplasms, cerebrospinal fluid, esophageal neoplasms, leptomeningeal carcinomatosis, neoplasm metastasis

## Abstract

Leptomeningeal carcinomatosis (LMC) is a rare but devastating complication of solid tumors. Involvement from esophageal cancer is exceedingly uncommon and poorly characterized, with evidence limited to case reports and small series. PubMed/MEDLINE, Scopus, Embase, Web of Science, and the Cochrane Library were systematically searched by two investigators for LMC originating from esophageal cancer. Thirty-one studies reporting 43 patients were included (93% male; mean age 59.3 years). Esophageal adenocarcinoma was the predominant histology (86%), followed by squamous cell carcinoma (11.6%), most commonly from the distal esophagus or esophagogastric junction. LMC occurred synchronously in 27.9% and metachronously in 72.1%, with a median interval of 11.2 months between primary diagnosis and leptomeningeal disease. At diagnosis, 37.2% had isolated leptomeningeal involvement without extracranial metastases. Presentation was heterogeneous, most frequently including headache or meningeal symptoms (55.8%), vestibulocerebellar manifestations (39.5%), and cranial nerve involvement (37.2%). Cerebrospinal fluid cytology was positive in 81.1% of tested cases, often requiring repeat lumbar punctures. Molecular data were reported for 27.9% of patients; Human Epidermal Growth Factor Receptor 2 (HER2) was positive in 3 of 10 tested cases (30%). Management was individualized: radiotherapy in 37.2%, intrathecal therapy in 20.9%, and supportive care alone in 46.5%. Median survival was 1.1 months, though three patients achieved 12, 14, and 19 months following Central Nervous System (CNS)-directed therapy. LMC from esophageal cancer is rare and clinically aggressive, characterized by heterogeneous presentation, frequent diagnostic delay, and poor prognosis. Early recognition and palliative care are essential, while rare long-term survivors suggest selected patients may benefit from individualized CNS-directed treatment.

## Graphical Abstract

**Graphical Abstract:**
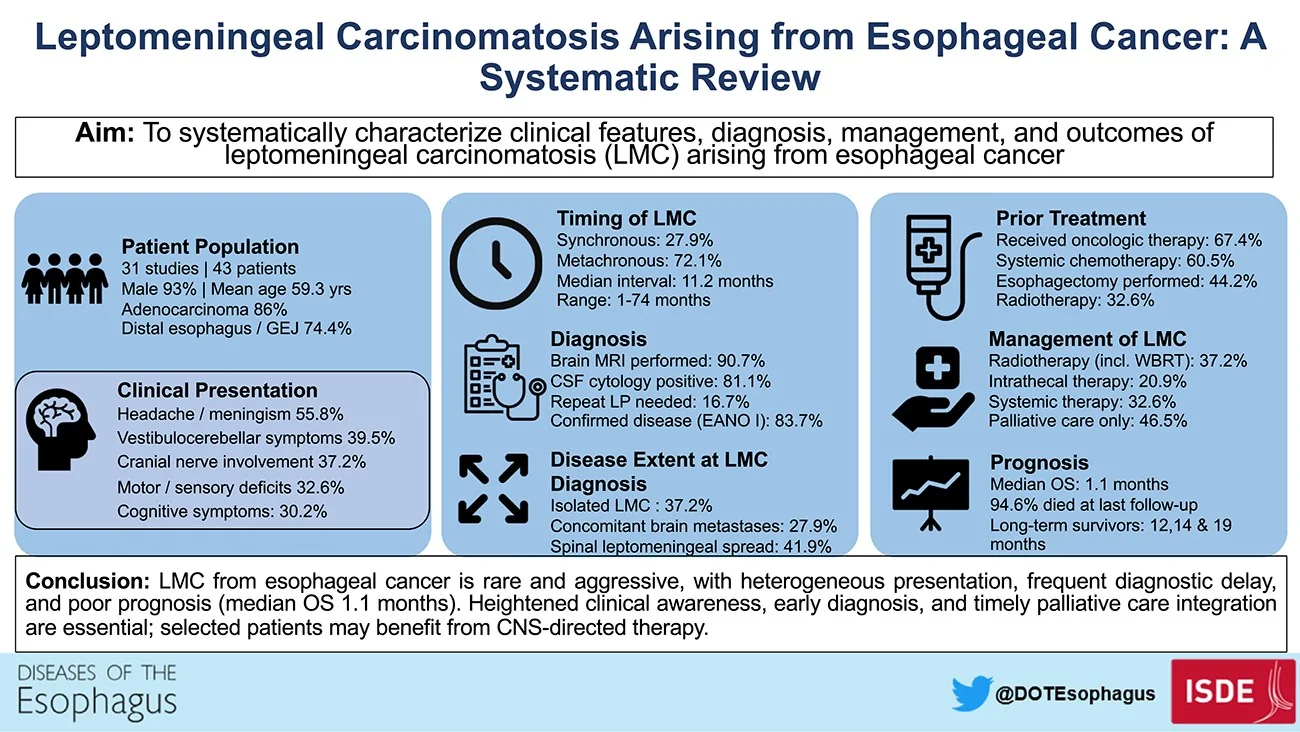

## INTRODUCTION

Leptomeningeal carcinomatosis (LMC) represents a devastating form of metastatic disease, characterized by diffuse infiltration of the leptomeninges and cerebrospinal fluid (CSF) by malignant cells.^1^ It occurs in approximately 3–8% of patients with advanced solid tumors and is most commonly associated with breast cancer, lung cancer, and melanoma.^2^ Clinically, LMC is characterized by heterogeneous neurological manifestations, significant diagnostic challenges, and a dismal prognosis, with survival often ranging from weeks to a few months despite best available treatment.^3^

Leptomeningeal involvement arising from esophageal cancer is exceedingly rare. Esophageal and gastroesophageal junction cancers predominantly metastasize to locoregional lymph nodes, liver, lung, and bone, while central nervous system involvement is uncommon and typically limited to parenchymal brain metastases in advanced disease.^4^^,^^5^ As a result, LMC originating from esophageal cancer remains poorly characterized, with existing knowledge largely confined to isolated case reports and small case series.^6^ This rarity, combined with nonspecific neurological presentations and limited clinician awareness, contributes to frequent diagnostic delay and uncertainty regarding optimal management.^6^

The aim of this systematic review was to synthesize all published cases of LMC arising from esophageal cancer, in order to characterize patient demographics, tumor features, timing of leptomeningeal involvement, clinical presentation, diagnostic approaches, management strategies, and survival outcomes. By integrating the available evidence, this review aims to enhance clinical recognition, inform diagnostic and therapeutic decision-making, and identify gaps in knowledge warranting further investigation in this rare but devastating manifestation of esophageal cancer.

## METHODS

This systematic review was conducted in accordance with the Preferred Reporting Items for Systematic Reviews and Meta-Analyses guidelines.^7^ The study protocol was registered prospectively in the International Prospective Register of Systematic Reviews (PROSPERO) database (Registration ID: CRD4202510457). A literature search was independently performed by two investigators using the PubMed/MEDLINE, Scopus, Embase, Web of Science, and Cochrane databases on LMC originating from esophageal cancer (last search performed on 5 July 2026), using the following algorithm: (“esophageal” OR “esophagus”) AND (“leptomeningeal” OR “leptomeningeal carcinomatosis” OR “meningeal carcinomatosis” OR “malignant meningitis” OR “neoplastic meningitis”). In addition to database searching, the reference lists of all eligible articles, as well as relevant narrative reviews and case series, were manually screened to identify additional studies. Any controversies were adjudicated via the decision of a senior investigator.

All articles written in the English language presenting case reports or case series were included in this systematic review, whereas reviews and meta-analyses were excluded. In addition, articles with an unclear diagnosis or insufficient clinical data were also excluded. Large retrospective institutional cohorts that did not report extractable individual-patient data were not eligible for the pooled patient-level synthesis, because individual-patient variables could not be abstracted from aggregate reporting. Where a report described patients with both esophageal or esophagogastric junction primaries and gastric primaries, only the patients with an esophageal or junctional primary were extracted, and the gastric cases were excluded at the patient level. Tumors described as arising predominantly within the stomach were not eligible, since an esophageal origin could not be established. Because the Siewert classification was not reported in any of the included studies, esophagogastric junction tumors could not be subtyped on the basis of the primary reports alone. For the subset of patients who underwent resection, the operative approach was therefore used as a supporting criterion for anatomical assignment, on the basis that the procedure chosen reflects the operating surgeon’s assessment of the tumor epicenter. Patients treated by total gastrectomy were regarded as having cardia cancer and were excluded, whereas patients treated by esophagectomy, including Ivor–Lewis esophagectomy, were regarded as having esophageal cancer and were included.

Data extraction was performed independently by two authors using a predefined and standardized data collection form. Extracted variables included demographic characteristics, tumor histology and primary tumor location, neurological clinical presentation, diagnostic modalities and method of LMC confirmation, presence of concomitant brain parenchymal metastases and other systemic metastases, timing of LMC in relation to the primary diagnosis, prior oncologic treatments, therapeutic approaches for the primary tumor, systemic disease and leptomeningeal disease, duration of follow-up, survival after LMC diagnosis, and patient outcomes. Available molecular and biomarker data were also extracted, including HER2 status and the assay used (immunohistochemistry, *in situ* hybridization, or next-generation sequencing), Programmed Death-Ligand 1 (PD-L1) combined positive score, mismatch-repair or microsatellite-instability status, and any reported genomic sequencing results; HER2 positivity was expressed as a proportion of tested cases rather than of the whole cohort, given the high proportion of untested patients. Cases were additionally classified according to the 2023 European Association of Neuro-Oncology (EANO) - European Society for Medical Oncology (ESMO) framework for leptomeningeal metastasis (types I/II by cytological or histological confirmation, and imaging subtypes A–D) wherever the reported data permitted.^8^ The complete patient-level dataset is provided in Supplementary Table S1.

Subgroup analyses compared survival by tumor histology (adenocarcinoma vs. squamous cell carcinoma [SCC]) and by primary tumor site (non-junctional esophageal vs. esophagogastric junction), using the log-rank test. Sensitivity analyses were performed by separating synchronous from metachronous presentations and those who received any CNS-directed therapy from those who received supportive care alone. Given the small number of patients in several strata and the descriptive nature of the underlying evidence, all subgroup and sensitivity analyses were regarded as hypothesis-generating rather than confirmatory, and non-significant comparisons are not interpreted as demonstrating equivalence.

The methodological quality and risk of bias of the included studies were independently appraised by two reviewers using the Joanna Briggs Institute (JBI) critical appraisal checklists—the 8-item checklist for case reports and the 10-item checklist for case series.^9^ Each item was rated as “yes,” “no,” “unclear,” or “not applicable,” and disagreements were resolved by a senior investigator. The item-level results are provided in Supplementary Table S2.

Numerical variables are presented as mean ± standard deviation or median (range), whereas categorical variables are described as frequency and percentages. Patients included in case series were considered as unique cases in order to estimate the variables of interest. Survival analysis was performed using the Kaplan–Meier method. Due to the lack of some required information in several studies, rates were calculated based on the available data. Statistical analysis was conducted using IBM SPSS Statistics for Windows, Version 29.0 (IBM Corp, NY).

## RESULTS

This systematic review includes 31 studies, reporting on a total of 43 patients with LMC from esophageal cancer.^6^^,^^10–39^ A detailed trial flowchart is presented in Figure 1. Among the included studies, 26 were individual case reports and 5 were case series. Of the 43 patients, 3 were female (7%) and 40 were male (93%), yielding a male-to-female ratio of 13:1. The mean age at presentation was 59.3 ± 10.1 years.

**Fig. 1:**
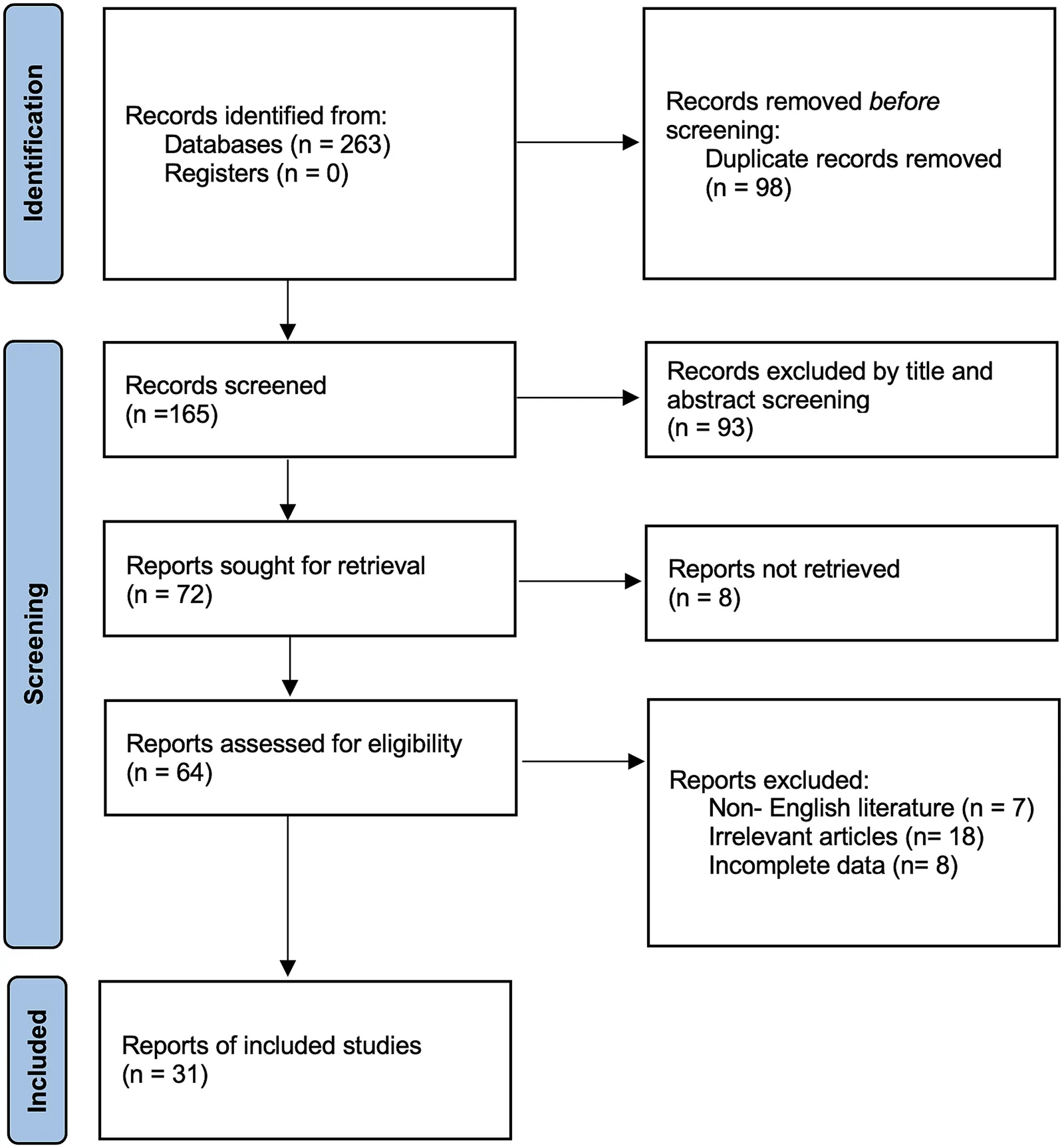
Trial flow of this review.

Among the 43 patients included in the analysis, the predominant histologic subtype was esophageal adenocarcinoma, identified in 37 patients (86%), while SCC, including one basaloid (squamous) variant, was reported in 5 patients (11.6%); the histologic subtype was not specified in 1 patient (2.3%). Among the 34 patients in whom the site was reported, the esophagogastric junction was involved in 23 (53.5% of the whole cohort) and the tumor was non-junctional esophageal in 11 (25.6%).

Synchronous LMC, defined as leptomeningeal involvement detected at the time of initial esophageal cancer diagnosis or during baseline staging prior to initiation of oncologic treatment, was observed in 12 of 43 patients (27.9%). The remaining 31 patients (72.1%) developed metachronous LMC during their disease. Among patients with metachronous LMC, the median interval from primary esophageal cancer diagnosis to LMC development was 11.2 months, with a reported range of 1–74 months.

At the time of leptomeningeal metastasis diagnosis, extracranial metastatic disease was present in 26 of 43 patients (60.5%), whereas 16 patients (37.2%) had no documented extracranial metastases, representing cases of isolated leptomeningeal involvement; the extent of systemic disease was not reported in 1 patient (2.3%). The most frequently reported extracranial metastatic sites included the lymph nodes, liver, bone, lung, adrenal glands, and pleural involvement, often in combination. Concomitant brain parenchymal metastases were identified in 12 of 43 patients (27.9%), most commonly involving the brainstem, cerebellum, cerebral hemispheres, and spinal cord, including cases with associated vertebral and spinal cord extension at the time of leptomeningeal metastasis diagnosis.

Prior oncologic therapy before the diagnosis of leptomeningeal metastasis was administered in 29 of 43 patients (67.4%), while 14 patients (32.6%) had received no oncologic treatment before LMC diagnosis. Systemic therapy (chemotherapy with or without radiotherapy) had been administered in 26 patients (60.5%), encompassing neoadjuvant, adjuvant, definitive, or palliative regimens. Radiotherapy directed at the primary tumor, either as part of chemoradiation or combined with systemic chemotherapy, was reported in 14 patients (32.6%). Surgical resection of the primary tumor, predominantly esophagectomy, had been performed in 19 patients (44.2%), most commonly in combination with perioperative systemic treatment.

At the time of leptomeningeal metastasis diagnosis, patients most commonly presented with headache and meningeal symptoms, reported in 24 of 43 cases (55.8%), frequently accompanied by nausea and vomiting. Vestibulocerebellar manifestations, including dizziness, vertigo, gait instability, and ataxia, were observed in 17 patients (39.5%). Cranial nerve involvement was prominent, occurring in 16 patients (37.2%), with hearing loss, diplopia, dysphagia, dysarthria, and visual impairment among the most frequent findings. Motor and sensory deficits were present in 14 patients (32.6%), altered mental status or cognitive symptoms in 13 patients (30.2%), ranging from confusion and cognitive decline to delirium and reduced level of consciousness, and seizures in 8 patients (18.6%). A detailed overview of patient clinical presentation is provided in Table 1.

**Table 1:** Clinical presentation in patients with esophageal leptomeningeal metastasis at the time of the diagnosis reported in the literature

**Symptom category**	**Clinical manifestations**	**Patients, *n* (%)**
Headache/meningeal symptoms	Headache, neck pain, meningism, photophobia	24 (55.8)
Vestibulocerebellar symptoms	Dizziness, vertigo, gait instability, ataxia, imbalance, nystagmus, falls, syncope	17 (39.5)
Cranial nerve involvement	Hearing loss (sudden/progressive), tinnitus, diplopia, oscillopsia, facial numbness or palsy, ptosis, hoarseness, dysphagia, dysarthria, visual loss, bilateral blindness	16 (37.2)
Motor and sensory deficits	Generalized weakness, focal limb weakness, hemiparesis, quadriparesis, paresthesia	14 (32.6)
Altered mental status/cognitive symptoms	Confusion, cognitive decline, delirium, agitation, hallucinations, fluctuating consciousness, lethargy, decreased level of consciousness	13 (30.2)
Gastrointestinal/systemic symptoms	Nausea, vomiting, weight loss, fatigue, fever	13 (30.2)
Spinal/radicular symptoms	Back pain, neck pain, saddle anesthesia, urinary retention, fecal incontinence	8 (18.6)
Seizures	Focal seizures, generalized convulsions, loss of consciousness	8 (18.6)
Other presentations	Painless enlarging scalp mass	1 (2.3)
Asymptomatic	Detected on surveillance imaging	1 (2.3)

Note: Percentages may exceed 100% as patients could present with multiple symptoms.

Radiological imaging at the time of leptomeningeal metastasis diagnosis most commonly consisted of brain magnetic resonance imaging (MRI), which was performed in 39 of 43 patients (90.7%), frequently combined with MRI of the spine (19 patients, 44.2%), while Computed Tomography (CT) was used either as an initial or adjunct modality in 15 patients (34.9%). Spinal leptomeningeal involvement, including enhancement of the spinal cord surface, nerve roots, or cauda equina, was documented in 18 patients (41.9%). Cranial nerve involvement, most commonly affecting the acoustic and trigeminal nerves and the internal auditory canals, was reported in nine patients (20.9%). Hydrocephalus or ventriculomegaly was identified in four patients (9.3%).

Diagnosis was primarily based on CSF analysis. CSF cytology results were available for 37 patients, among whom 30 cases (81.1%) demonstrated positive cytology and 7 cases (18.9%) had negative findings. Among the 30 patients with positive CSF cytology, diagnostic confirmation was achieved on the first lumbar puncture in 25 patients (83.3%), whereas 5 patients (16.7%) required repeat CSF sampling, with up to four lumbar punctures performed to obtain cytological confirmation.^15^^,^^17^^,^^21^^,^^25^ In cases without cytological confirmation, diagnosis was established on the basis of characteristic neuroradiological findings. In addition to cytology, histopathological confirmation was obtained in three patients (7.0%) through leptomeningeal biopsy, surgical biopsy of an intradural lesion, or resection of a meningo-cerebral lesion,^12^^,^^34^ and in a further two patients the diagnosis was made at post-mortem examination.^19^^,^^31^ In one patient, quantitative reverse-transcription Polymerase Chain Reaction (PCR) for carcinoembryonic antigen (CEA) Messenger Ribonucleic Acid (mRNA) in the CSF, combined with cytokeratin immunostaining, established the diagnosis when both cytology and imaging were non-diagnostic.^30^

When the cohort was retrospectively classified according to the 2023 EANO–ESMO criteria, 36 of 43 patients (83.7%) met the definition of confirmed (type I) leptomeningeal metastasis: 30 on the basis of positive CSF cytology, 3 by leptomeningeal, intradural, or surgical biopsy, 2 at post-mortem examination, and 1 by molecular and immunocytochemical detection of tumor cells in CSF. The remaining seven patients (16.3%) were classified as probable or possible (type II) on the basis of characteristic clinical and neuroimaging findings without cytological or histological confirmation.

With respect to imaging subtype, a linear enhancement pattern (type A) was most common (15 patients, 34.9%), followed by nodular disease (type B) in 11 patients (25.6%) and combined linear and nodular disease (type C) in 6 patients (14.0%); 7 patients (16.3%) had no diagnostic leptomeningeal abnormality on initial imaging or hydrocephalus as the sole finding (type D), and the imaging pattern could not be classified from the published description in 4 patients (9.3%).

Following the diagnosis of leptomeningeal metastasis, systemic oncologic treatment was administered in 14 of 43 patients (32.6%), most commonly in the form of systemic chemotherapy, either alone or in combination with targeted or immunotherapeutic agents. Treatment directed specifically at leptomeningeal disease varied considerably. Radiotherapy was employed in 16 patients (37.2%), most frequently as whole-brain radiotherapy, occasionally combined with focal spinal or skull-base irradiation. Intrathecal therapy was administered in 9 patients (20.9%), including intrathecal methotrexate, thiotepa, topotecan, liposomal cytarabine, and intrathecal trastuzumab, sometimes in combination with radiotherapy. Overall, 20 patients (46.5%) received some form of CNS-directed therapy (radiotherapy, intrathecal therapy, or both). In contrast, best supportive or palliative care alone, including corticosteroids or symptomatic management without disease-directed therapy, was reported in 20 patients (46.5%) at the time of leptomeningeal metastasis diagnosis. Lastly, the treatment approach was not reported in two patients (4.7%).

Tumor molecular or biomarker data of any kind were reported in 12 of 43 patients (27.9%), and the panel of markers assessed varied considerably between reports as presented in Table 2. HER2 was the most frequently documented biomarker, evaluated in 10 patients (23.3%). Among tested cases, HER2 positivity was identified in 3 of 10 patients (30%), while 7 of 10 (70%) were HER2-negative. The assay used differed across reports: immunohistochemistry was performed in five patients, fluorescence *in situ* hybridization in five, and next-generation sequencing in two. Other biomarkers were reported less often. PD-L1 was assessed in two patients (4.7%), with combined positive scores of 5 and 100, and mismatch-repair or microsatellite-instability status in four patients (9.3%), all of whom were mismatch-repair proficient. Broad next-generation sequencing panels were reported in four patients (9.3%);Tumor Protein 53 (TP53) alterations were present in all four, Cyclin Dependent Kinase 6 (CDK6) amplification in two, and Cyclin E1 (CCNE1) co-amplification in one HER2-amplified tumor. In a single additional patient, quantitative reverse-transcription PCR for CEA mRNA in CSF established the diagnosis when cytology and imaging were both non-diagnostic, and the CEA mRNA score fell in parallel with clinical improvement during systemic chemotherapy.

**Table 2:** Reported molecular and biomarker status across patients with esophageal leptomeningeal metastasis from esophageal cancer reported in the literature

**Biomarker/assay**	**Tested, *n* (% of 43)**	**Result**
HER2	10 (23.3%)	Positive 3/10 (30.0%); negative 7/10 (70.0%)
By immunohistochemistry	5 (11.6%)	1 positive (IHC 3+); 1 equivocal (IHC 2+, ISH-negative); 3 negatives
By FISH/ISH	5 (11.6%)	0 amplified
By next-generation sequencing	2 (4.7%)	2 ERBB2-amplified (one FISH-negative)
PD-L1 (combined positive score)	2 (4.7%)	CPS 5 and CPS 100
Mismatch repair/MSI	4 (9.3%)	4 proficient (pMMR/MSS); 0 deficient
Broad NGS panel	4 (9.3%)	TP53 altered in 4/4; CDK6 amplified in 2; CCNE1 co-amplified in 1
CSF CEA mRNA (RT-PCR)	1 (2.3%)	Positive; used to establish the diagnosis and to monitor response

CPS, combined positive score; CSF, cerebrospinal fluid; FISH, fluorescence *in situ* hybridization; IHC, immunohistochemistry; ISH, *in situ* hybridization; MSI, microsatellite instability; MSS, microsatellite stable; NGS, next-generation sequencing; pMMR, mismatch-repair proficient.

Note: Assay categories are not mutually exclusive, as some patients underwent more than one HER2 assay

Two of the three patients with prolonged survival after leptomeningeal diagnosis harbored a potentially actionable biomarker: one had HER2-amplified disease treated with intrathecal trastuzumab (19 months), and the other had a PD-L1 combined positive score of 100 treated with intrathecal topotecan and systemic checkpoint blockade (14 months). The third long-term survivor, who lived 12 months on intrathecal methotrexate-based therapy, underwent no molecular testing at all.

Follow-up and survival data after the diagnosis of leptomeningeal metastasis were reported in 37 of 43 patients. The median survival was 1.1 months, with a reported range from 0.1 to 19 months. At last follow-up, 35 of the 37 patients with reported follow-up had died, while 2 patients were alive, with reported survivals of 14 and 19 months, respectively. Overall survival after the diagnosis of leptomeningeal metastasis is illustrated in Figure 2.

**Fig. 2:**
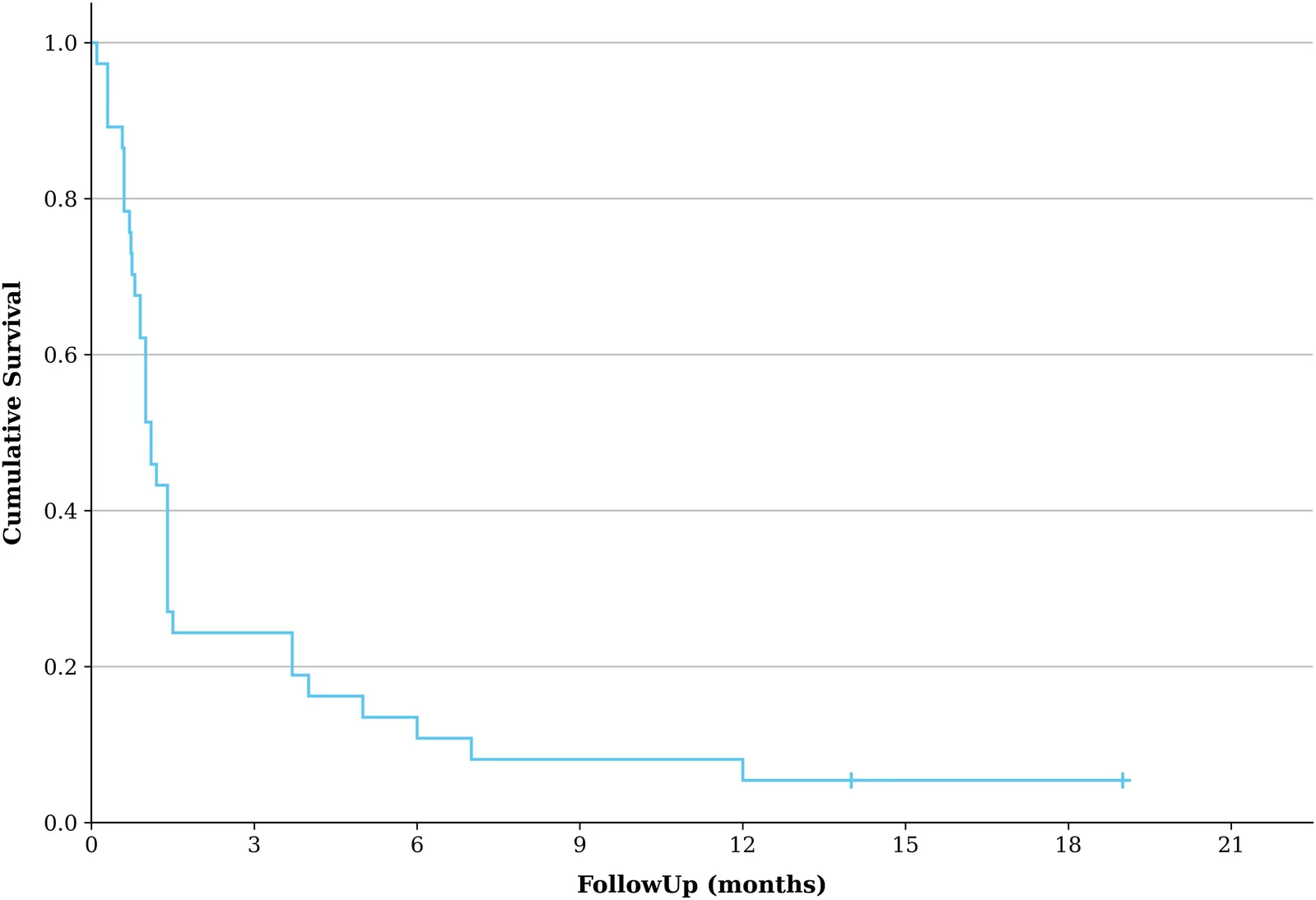
Kaplan–Meier overall survival curve after diagnosis of leptomeningeal metastasis.

On subgroup analysis by histology, median survival was 1.1 months in patients with adenocarcinoma and 1.0 month in those with SCC (log-rank *P* = 0.56). This comparison must be interpreted with great caution: only three of the five patients with SCC had quantified survival data, and the analysis is therefore substantially underpowered and cannot exclude a clinically meaningful difference.

Analysis by primary tumor site was constrained by the source literature. The Siewert classification was not reported in any of the 43 patients, and the esophageal segment of origin was not specified at all in 9 patients (20.9%). Among the 34 patients in whom the site could be assigned, median survival was 1.0 month for junctional and 1.4 months for non-junctional esophageal disease (log-rank *P* = 0.90).

Lastly, in an exploratory comparison, patients who received any CNS-directed therapy (radiotherapy, intrathecal therapy, or both) survived longer than those managed with supportive care alone (median 1.4 vs. 0.9 months, *P* < 0.001); this association is confounded by selection on performance status, neurological deficit, and extracranial disease burden, and by immortal time bias, since patients must survive long enough to be treated. The subgroup and sensitivity analyses are summarized in Table 3.

**Table 3:** Subgroup and sensitivity analyses of survival after diagnosis of LMC

**Comparison**		**Patients, *n***	**With quantified survival, *n***	**Median survival after LMC diagnosis (months)**	**Log-rank *P***
*Subgroup analyses*					
By histology	Adenocarcinoma	37	33	1.1	0.56^*^
	SCC	5	3	1.0	
By primary site location	Esophagogastric junction	23	19	1.0	0.90
	Esophageal (non-junctional)	11	9	1.4	
*Sensitivity analyses*					
By time of presentation	Synchronous LMC	12	9	1.0	0.93
	Metachronous LMC	31	28	1.1	
CNS-directed therapy	Any CNS-directed therapy	20	19	1.4	<0.001^**^
	Supportive care alone	20	18	0.9	

^*^Only three of the five patients with SCC had quantified survival data; the log-rank comparison is presented for completeness but is severely underpowered and should not be interpreted as evidence of equivalence.

^**^The comparison of CNS-directed therapy with supportive care alone is confounded by selection on performance status and by immortal time bias, since patients must survive long enough to receive treatment; it is presented as exploratory only.

Among the 31 included studies, 26 were individual case reports and 5 were case series; because several series reported their patients in sufficient individual detail, the risk-of-bias appraisal was performed at the level of the individual case for 30 patient reports and at the study level for the 2 remaining series. On critical appraisal using the JBI checklists, the 30 case reports achieved a median score of 7 of 8 (range 4–8), with 25 of 30 scoring 7 or 8. The item most frequently unmet was the reporting of adverse or unanticipated events, which was absent in 18 reports and unclear in a further one. The two case series scored 5 and 8 of 10, respectively, and were most often limited on the consecutive and complete inclusion of participants, which was unclear in both. Overall, the included evidence was judged to be at moderate-to-high risk of bias, consistent with its derivation from uncontrolled, case-level reports. No study was excluded on the basis of the appraisal, and the results are provided in Supplementary Table S2.

## DISCUSSION

LMC is a rare but devastating manifestation of metastatic cancer, occurring in approximately 3–8% of patients with advanced solid tumors and most frequently associated with breast cancer, lung cancer, and melanoma.^2^ In contrast, leptomeningeal involvement from esophageal and gastroesophageal junction adenocarcinoma is exceedingly uncommon and remains poorly characterized.^6^ By systematically synthesizing all available published cases, the present review provides the most comprehensive overview to date of the clinical characteristics, diagnostic approach, management strategies, and outcomes of esophageal cancer-associated LMC.

The pathogenesis of LMC in esophageal cancer remains incompletely understood, but available evidence suggests that it is not simply a consequence of advanced systemic disease burden. In this systematic review, more than one-third of the patients (37.2%) had isolated LMC associated with primary esophageal cancer. The frequent absence of concomitant visceral or parenchymal brain metastases in reported cases supports the hypothesis of possible early hematogenous dissemination by a biologically distinct subpopulation of tumor cells with specific tropism for the leptomeningeal compartment.^40^ Proposed routes of entry include traversal of the blood–CSF barrier, particularly via the fenestrated capillaries of the choroid plexus, or passage through the arachnoid–CSF interface.^40^^,^^41^ Successful leptomeningeal colonization likely requires tumor cells to possess specialized molecular characteristics that enable both penetration of tight-junction barriers and survival within the pauci-cellular, low-nutrient CSF microenvironment.^42^ The occurrence of leptomeningeal disease in patients with limited primary tumor burden further suggests that specific tumor clones may exploit anatomical corridors to access the central nervous system sanctuary independently of widespread systemic dissemination.^40^ Together, these observations support a model in which esophageal cancer-associated LMC represents a biologically selective process rather than a passive consequence of advanced disease.

The clinical presentation of esophageal cancer-associated LMC is notably heterogeneous but demonstrates recurrent and possibly clinically meaningful neurological patterns. Rather than presenting with a single dominant syndrome, patients frequently exhibited multifocal neurological involvement, reflecting diffuse dissemination along the neuraxis.^43^ Prominent clinical phenotypes included cranial nerve–dominant presentations, particularly involving the vestibulocochlear and trigeminal nerves, manifesting as hearing loss, vertigo, diplopia, and facial sensory disturbances. This pattern is consistent with prior observations in leptomeningeal metastasis from solid tumors, in which tumor cells preferentially involve the posterior fossa, basal cisterns, and perineural spaces.^41^ A second common pattern was characterized by meningeal irritation and encephalopathy, including headache, nausea, vomiting, hydrocephalus, altered mental status, and fluctuating levels of consciousness. These presentations may mimic infectious or inflammatory meningitis and are a well-recognized contributor to diagnostic delay in leptomeningeal disease.^18^^,^^44^^,^^45^ Additionally, spinal and cauda equina-predominant presentations, such as back pain, lower limb weakness, sensory deficits, and sphincter dysfunction, were observed and likely reflect extensive leptomeningeal involvement of the spinal cord and nerve roots, a distribution pattern consistently described in solid tumors with CSF dissemination.^33^ These recurrent neurological patterns have important implications for clinical recognition and diagnostic suspicion. Therefore, LMC should be suspected in patients with esophageal cancer who develop acute or subacute neurological symptoms that are otherwise unexplained, particularly sudden or progressive bilateral hearing loss, subacute vertigo accompanied by headache, or features of communicating hydrocephalus. Additional red flags include the presence of multiple cranial neuropathies in the absence of parenchymal brain metastases and persistent or progressive neurological deterioration despite negative initial CSF cytology.

Diagnosis of LMC in our review was frequently delayed and challenging. Despite advances in neuroimaging and CSF analysis, both MRI and CSF cytology demonstrate limited sensitivity at initial evaluation.^46^ False-negative MRI studies and negative initial CSF cytology were common, necessitating repeat lumbar punctures, repeat imaging, or alternative diagnostic approaches.^15^^,^^17^^,^^21^^,^^25^ In selected cases, diagnosis was ultimately established through leptomeningeal or intradural biopsy.^12^ These findings highlight the limitations of conventional diagnostic tools and reinforce the importance of maintaining a high index of suspicion in patients with compatible neurological symptoms, even in the setting of initially negative investigations.

The timing of leptomeningeal involvement further underscores the distinct biology and clinical behavior of this condition. In the cases reviewed, LMC developed both synchronously at initial esophageal cancer diagnosis and metachronously after highly variable intervals, extending to up to 74 months from initial diagnosis or curative intent therapy.^6^^,^^15^^,^^27^^,^^28^^,^^34^ Importantly, several patients experienced isolated leptomeningeal relapse in the absence of active extracranial disease, and this patterns highlight that leptomeningeal involvement can represent a temporally unpredictable manifestation of esophageal cancer with substantial implications for clinical vigilance, longitudinal follow-up, and recognition of new neurological symptoms even years after primary treatment.

Management of esophageal cancer-associated LMC was highly individualized and predominantly palliative in intent. Treatment strategies varied widely and included systemic therapy, intrathecal chemotherapy or targeted agents, radiotherapy, and supportive care alone, reflecting the heterogeneity of clinical presentations, disease burden, and patient performance status, precluding the definition of a standardized management strategy. Overall prognosis remains extremely poor, with survival after LMC diagnosis in this review typically measured in weeks to a few months and rapid neurological deterioration observed in the majority of patients, consistent with outcomes reported for leptomeningeal metastasis from other solid tumors.^47–49^ However, three patients in our review represented clear exceptions to this pattern, achieving prolonged survival of 12, 14, and 19 months. All three received CNS-directed treatment: whole-brain radiotherapy with focal spinal radiotherapy combined with intrathecal topotecan and systemic checkpoint blockade in one case; intrathecal trastuzumab administered in combination with radiotherapy in another; and intrathecal methotrexate, escalated to methotrexate with cytarabine and dexamethasone, in the third.^12^^,^^27^^,^^35^ While such outcomes remain uncommon and cannot be generalized, these exceptional cases suggest that selected patients may derive meaningful benefit from intensive, targeted intrathecal and radiotherapeutic approaches. Collectively, these findings underscore the importance of early diagnosis, realistic prognostication, and timely integration of supportive and palliative care, alongside careful consideration of disease-directed interventions in carefully selected patients.

The heterogeneity of treatment observed across these reports largely reflects the era in which they were published, most predated, any dedicated guidance for leptomeningeal metastasis. Contemporary practice is now framed by the 2023 EANO–ESMO Clinical Practice Guideline, which stratifies management according to a combined clinical, cytological (type I/II) and neuroimaging (type A–D) classification. It recommends best supportive care for patients with poor performance status and an anticipated survival of less than 1 month, and reserves central nervous system-directed therapy—intrathecal pharmacotherapy, focal or craniospinal radiotherapy, and molecularly targeted or systemic agents with central nervous system penetration—for selected patients with better performance status.^8^ In parallel, the National Comprehensive Cancer Network (NCCN) Central Nervous System Cancers guideline applies a “good-risk” versus “poor-risk” dichotomy: good-risk patients—those with adequate performance status, no major neurological deficits, limited systemic disease and available systemic options—are candidates for active multimodality treatment, including intrathecal therapy and craniospinal irradiation, whereas poor-risk patients are directed to palliative care.^50^ Both converge on the same principle: the decision to treat is based on the patient status and the burden of the disease outside of the neuraxis.

Our case-level synthesis should be read alongside the largest institutional experience reported to date, in which 20 of 3283 patients (0.61%) with gastroesophageal carcinoma treated at the Princess Margaret Cancer Centre developed LMC.^51^ That cohort was not incorporated into our pooled analysis because it reports aggregate rather than extractable individual-patient outcomes, but its findings are related to our own. Most importantly, both identify a survival advantage with central nervous system-directed radiotherapy: whole-brain radiotherapy was associated with a median survival of 2.8 months versus 0.7 months with best supportive care in their series, and radiotherapy with 1.4 months versus 0.9 months with supportive care alone in ours (*P* < 0.001). The survival benefit was smaller in our cohort and both arms sit lower. This consistent downward shift is better explained by later recognition: diagnosis in the published case literature was frequently made only after fixed neurological deficits had developed, so patients entered observation later in their disease course than in a systematically followed institutional cohort. Taken together, they suggest that radiotherapy retains a role in patients whose functional status permits it, even where the expected survival gain is measured in weeks, though neither comparison excludes confounding by performance status. Molecular characterization, by contrast, was too sparse in either dataset to permit meaningful comparison: HER2 was positive in 2 of 13 tested patients (15%) in their series and in 3 of our 10 (30.0%); a small sample size to allow interpretation. Beyond HER2, biomarker reporting was almost entirely absent; PD-L1 was assessed in two patients and mismatch-repair status in four across our cohort. The two datasets are complementary rather than duplicative: their institutional design provides the only incidence estimate available in this disease (0.61% of 3283 patients with gastroesophageal carcinoma), while our synthesis documents the clinical course of each patient: isolated leptomeningeal disease in 37.2%, intervals from primary diagnosis to leptomeningeal disease extending to 74 months, and durable responses in selected patients both with and without an actionable target.

This systematic review provides the most comprehensive synthesis to date of LMC arising from esophageal cancer, a rare and poorly characterized clinical entity; however, several limitations should be acknowledged. The available evidence is derived exclusively from case reports and small case series, introducing inherent risks of publication bias, selective reporting, and heterogeneity in data quality. A formal risk-of-bias appraisal using the JBI tools confirmed that the evidence base is at moderate-to-high risk of bias, and the pooled estimates should be interpreted accordingly. Due to the limited number of reported cases and substantial heterogeneity in clinical, radiological, and therapeutic data, no reliable assumptions can be made regarding patient outcomes. In addition, cases of LMC may have been underrecognized or misattributed to nonspecific neurological deterioration or overall disease progression, particularly in the absence of definitive diagnostic confirmation.

The boundary between esophageal, junctional, and gastric primaries is imperfectly drawn in the source literature, and two of the reports identified in this review described esophageal or junctional cases alongside gastric ones; in these instances, we extracted only the patients with an esophageal or junctional primary. This is complicated by the fact that the Siewert classification, which exists precisely to resolve such ambiguity and which the Princess Margaret investigators applied, was not reported in a single one of our 43 patients, and in 9 (20.9%) even the esophageal segment of origin was unstated. In its absence we used the operative approach as an indication in resected patients: total gastrectomy was taken to indicate a cardia tumor and led to exclusion, whereas esophagectomy, including Ivor–Lewis esophagectomy, was taken to indicate an esophageal tumor and led to inclusion. Subsequently, no patient in the final cohort had undergone total gastrectomy. The limitations of this assumption are evident: operative approach is influenced by institutional practice, surgeon preference and era as well as by anatomy, and it could be applied to only the 17 patients (39.5%) in whom a resection was reported, leaving the remaining assigned on narrative description alone. The near-universal absence of Siewert subtyping in the published case literature is itself a substantive finding of this review and a reporting standard that future series should adopt.

Our subgroup analyses are limited by the small number of patients in several strata. In particular, only three of the five patients with SCC had quantified survival data, so the comparison by histology is underpowered, and the absence of a statistically significant difference should not be taken as evidence that the histologies behave similarly. The same handicap applies to histologic and molecular subtyping: a signet-ring component was documented in 9 patients (20.9%), but the variant went unreported in 11; HER2 was tested in fewer than a quarter of patients. These gaps limit any insights into potential biological drivers of leptomeningeal dissemination or treatment response, and the observed rates should be regarded as an estimate among a selected, tested minority rather than a prevalence figure. Karnofsky performance status was not reported in any included study, precluding retrospective application of the risk stratification on which both current guidelines depend. Finally, the restriction to English-language publications and the reliance on published case reports and small series mean that unpublished and negative cases are likely under-represented. Given the retrospective and descriptive nature of the included data, the associations reported here should be regarded as hypothesis-generating rather than as evidence of causation.

Therefore, future research should prioritize multicenter collaboration and patient registries to facilitate standardized reporting of clinical presentation, neuroimaging, CSF findings, treatment strategies, and outcomes in esophageal cancer-associated LMC, with performance status, histologic variant, and anatomical subsite recorded. Integration of molecular profiling, including CSF-based liquid biopsy approaches, may help elucidate the biological drivers of leptomeningeal dissemination and identify therapeutic targets. Finally, the rare durable responses support prospective evaluation of CNS-directed therapy in patients with isolated leptomeningeal disease and good clinical condition, in whom benefit appears most likely.

## CONCLUSION

LMC from esophageal cancer represents a rare and clinically aggressive manifestation with heterogeneous presentation, frequent diagnostic delay, and poor prognosis. This review highlights that leptomeningeal disease may occur as an isolated central nervous system relapse and long after apparently curative treatment, underscoring the need for heightened clinical awareness and timely diagnosis. Given the limited efficacy of available treatments, early integration of palliative and supportive care remains essential, while rare cases of prolonged survival suggest that selected patients may benefit from individualized, CNS-directed therapeutic approaches.

## Supplementary Material

Supplementary_material_doag126

## Notes

Specific author contributions: Adam Mylonakis (Conceptualization, Formal analysis, Investigation, Writing—original draft), Dimosthenis Stylios (Data curation, Formal analysis, Writing—original draft), Diamantis Tsilimigras (Formal analysis, Methodology, Visualization, Writing—review & editing), Georgios Charalampopoulos (Investigation, Methodology, Software), Ioannis Kotsantis (Data curation, Formal analysis, Writing—original draft), Michail Liontos (Data curation, Methodology, Resources, Writing—review & editing), Ioannis Georgakopoulos (Formal analysis, Software, Writing—original draft), Dimitrios Schizas (Conceptualization, Project administration, Supervision, Visualization, Writing—review & editing).

Financial support: This research received no external funding and was conducted without financial support from any public, commercial, or not-for-profit funding agency.

## References

[1] Ozair A, Wilding H, Bhanja D et al. Leptomeningeal metastatic disease: new frontiers and future directions. Nat Rev Clin Oncol 2025; 22: 134–54. 10.1038/s41571-024-00970-3. 39653782

[2] Nayar G, Ejikeme T, Chongsathidkiet P et al. Leptomeningeal disease: current diagnostic and therapeutic strategies. Oncotarget 2017; 8: 73312–28. 10.18632/oncotarget.20272. PMC5641214 29069871

[3] Sener U, Wilcox J A, Boire A A. Leptomeningeal disease: current approaches and future directions. Curr Neurol Neurosci Rep 2025; 25: 25. 10.1007/s11910-025-01412-y. PMC11920312 40100294

[4] Ai D, Zhu H, Ren W et al. Patterns of distant organ metastases in esophageal cancer: a population-based study. J Thorac Dis 2017; 9: 3023–30. 10.21037/jtd.2017.08.72. PMC5708381 29221275

[5] Hong H, Ding J, Yuan C et al. Incidence and prognosis of brain metastases in esophageal carcinoma: a systematic review and meta-analysis. J Thorac Dis 2025; 17: 8636–52. 10.21037/jtd-2025-1219. PMC12603509 41229813

[6] Lukas R V, Mata-Machado N A, Nicholas M K, Salgia R, Antic T, Villaflor V M. Leptomeningeal carcinomatosis in esophageal cancer: a case series and systematic review of the literature. Dis Esophagus 2015; 28: 772–81. 10.1111/dote.12276. 25142531

[7] Page M J, McKenzie J E, Bossuyt P M et al. The PRISMA 2020 statement: an updated guideline for reporting systematic reviews. BMJ 2021; 372: n71. 10.1136/bmj.n71. PMC8005924 33782057

[8] Rhun E L, Weller M, van den B M et al. Leptomeningeal metastasis from solid tumours: EANO–ESMO clinical practice guideline for diagnosis, treatment and follow-up. ESMO Open 2023; 8: 101624. 10.1016/j.esmoop.2023.101624. PMC10619142 37863528

[9] Munn Z, Barker T H, Moola S et al. Methodological quality of case series studies: an introduction to the JBI critical appraisal tool. JBI Evid Synth 2020; 18: 2127–33. 10.11124/JBISRIR-D-19-00099. 33038125

[10] Spricido I Y, Bignoto T C, Vazquez Y G, de Moraes P H Z. Complex diagnosis and management of metastatic esophagogastric junction adenocarcinoma with colonic and meningeal involvement. Einstein (São Paulo) 2025; 23: eRC1460. 10.31744/einstein_journal/2025RC1460. PMC12671620 41259497

[11] Grabill N, Louis M, Adams R, Gherasim C, So S, Stevens T. Leptomeningeal carcinomatosis in advanced esophageal adenocarcinoma: a case report on presentation and diagnosis. Int J Surg Case Rep 2025; 126: 110708. 10.1016/j.ijscr.2024.110708. PMC11667075 39642415

[12] Wu S A, Jia D T, Schwartz M et al. HER2+ esophageal carcinoma leptomeningeal metastases treated with intrathecal trastuzumab regimen. CNS Oncol. 2023; 12: CNS99. 10.2217/cns-2022-0018. PMC10410688 37219390

[13] Jagtap S V, Khoja S, Jagtap S S, Gudur R, Janugade H. Leptomeningeal carcinomatosis secondary to esophageal cancer diagnosed on cytology. J Neurosci Rural Pract 2020; 11: 495–7. 10.1055/s-0040-1713576. PMC7394655 32753822

[14] Lu Y, Wang L, Ajani J A. Rare esophageal leptomeningeal metastases detected on 18F-FDG PET/CT. Clin Nucl Med 2020; 45: 334–5. 10.1097/RLU.0000000000002952. 31977493

[15] Dumbill R, Thompson S, Peschl H, Turner G, Woodrow C. Isolated leptomeningeal carcinomatosis and possible fungal meningitis as late sequelae of oesophageal adenocarcinoma. BMJ Case Rep 2019; 12: e230117. 10.1136/bcr-2019-230117. PMC6827809 31678919

[16] Cunha R, Godinho J, Inácio M, Galriça N I. Palliative approach to leptomeningeal carcinomatosis in oesophagogastric junction cancer. BMJ Case Rep 2019; 12: e230114. 10.1136/bcr-2019-230114. PMC6803121 31604716

[17] Boursiquot R, Krol D, Hanif S, Rojas J, Jain M. Syndrome of inappropriate antidiuretic hormone in a patient with leptomeningeal carcinomatosis. J Med Case Rep 2016; 10: 73. 10.1186/s13256-016-0862-2. PMC4815213 27036107

[18] Ahmed M, Booth T, Otero A, Kelly I. Leptomeningeal carcinomatosis from oesophageal cancer, presenting as meningitis. BMJ Case Rep 2016; 2016: bcr2015210974. 10.1136/bcr-2015-210974. PMC4716441 26740266

[19] Adams M, Doherty C, O’Kane A et al. Malignant meningitis secondary to oesophageal adenocarcinoma presenting with sensorineural hearing loss: a series of three cases and discussion of the literature. Eur Arch Otorhinolaryngol 2016; 273: 2481–6. 10.1007/s00405-015-3842-z. 26645106

[20] Fresko B T, Selam M T, Tuzlali H, Batman B, Asoglu O. A fulminant case of leptomeningeal carcinomatosis secondary to esophageal adenocarcinoma. J Gastrointest Cancer 2016; 47: 111–3. 10.1007/s12029-015-9731-2. 25972065

[21] Lobo R, Pillay I, Kennedy B, Watts M. Confusion and altered behaviour? Cause. BMJ Case Rep 2013; 2013: bcr2012007960. 10.1136/bcr-2012-007960. PMC3603878 23341584

[22] Akhavan A, Navabii H. Leptomeningeal metastasis from squamous cell carcinoma of oesophagus with unusual presentation. BMJ Case Rep 2012; 2012: bcr0220125846. 10.1136/bcr-02-2012-5846. PMC4543016 23174999

[23] Aulakh A S, Buttar A, Piperdi B. Leptomeningeal carcinomatosis in esophageal cancer: case report and review of literature. J Gastrointest Cancer 2012; 43: 84–8. 10.1007/s12029-011-9334-5. 22038668

[24] Haynes R, Jones C, Loeser C. Initial presentation of esophageal adenocarcinoma with leptomeningeal carcinomatosis: 1606. Am J Gastroenterol 2016; 111: S753. 10.14309/00000434-201610001-01606.

[25] Astner S T, Nieder C, Stock K, Gaa J, Grosu A L. Carcinomatous meningitis appearing as acoustic neuromas: two cases. Strahlenther Onkol 2007; 183: 279–83. 10.1007/s00066-007-1615-3. 17497100

[26] Goddeeris K, Verslype C, Wilms G, Van Cutsem E. Leptomeningeal carcinomatosis associated with oesophageal adenocarcinoma: two case reports and review of the literature. Acta Gastroenterol Belg 2006; 69: 377–80. 17343079

[27] Kountourakis P, Papamichael D, Haralambous H et al. Leptomeningeal metastases originated from esophagogastric junction/gastric cancer: a brief report of two cases. World J Gastrointest Oncol 2018; 10: 56–61. 10.4251/wjgo.v10.i1.56. PMC5767794 29375749

[28] Wagemakers M, Verhagen W, van de Borne B, Venderink D, Wauters C, Strobbe L. Bilateral profound hearing loss due to meningeal carcinomatosis. J Clin Neurosci 2005; 12: 315–8. 10.1016/j.jocn.2004.07.012. 15851093

[29] Abdo A A, Coderre S, Bridges R J. Leptomeningeal carcinomatosis secondary to gastroesophageal adenocarcinoma: a case report and review of the literature. Can J Gastroenterol 2002; 16: 807–11. 10.1155/2002/659125. 12464975

[30] Okumura H, Natsugoe S, Yokomakura N et al. A case of leptomeningeal carcinomatosis from esophageal basaloid carcinoma diagnosed by quantitative reverse transcription-polymerase chain reaction for carcinoembryonic antigen. J Gastroenterol 2005; 40: 87–93. 10.1007/s00535-004-1498-3. 15692794

[31] Teare J P, Whitehead M, Rake M O, Coker R J. Rapid onset of blindness due to meningeal carcinomatosis from an oesophageal adenocarcinoma. Postgrad Med J 1991; 67: 909–11. 10.1136/pgmj.67.792.909. PMC2399160 1758802

[32] Mitera G, Roitman D, Pohani G et al. Leptomeningeal carcinomatosis secondary to gastroesophageal adenocarcinoma: a case report and literature review of a rare occurrence. Scholarly Res Exch 2009; 2009: 642154. 10.3814/2009/642154.

[33] Alkhotani A, Alrishi N, Alhalabi M S, Hamid T. Cauda equina syndrome secondary to leptomeningeal carcinomatosis of gastroesophageal junction cancer. Case Rep Neurol 2016; 8: 87–91. 10.1159/000445869. PMC4881252 27239185

[34] Na M K, Kim C H, Kim J M, Cheong J W, Ryu J I, Kim H W. Multiple meningocerebral metastasis and extensive skull metastasis from squamous cell carcinoma of esophagus: a case report and review of literature. Brain Tumor Res Treat 2016; 4: 142–4. 10.14791/btrt.2016.4.2.142. PMC5114187 27867927

[35] Pabon C M, Yeboa D N, O’Brien B J, Majd N K, Wang C, Murphy M A B. Intrathecal topotecan with systemic checkpoint inhibitor therapy for gastroesophageal cancer with leptomeningeal involvement: two case reports and review of the literature. J Gastrointest Oncol 2024; 15: 1331–40. 10.21037/jgo-24-70. PMC11231862 38989402

[36] Mishra S V . Leptomeningeal carcinomatosis as primary presentation in gastrointestinal (GI) malignancies - case series and review of literature. Clin Case Rep Int 2025; 9: 1731.

[37] Rizvi S, Fateh S, Abbi K K S, Jiang Y. Leptomeningeal carcinomatosis as the only manifestation of disease in recurrent gastroesophageal cancers. Gastrointest Cancer Res 2011; 4: 68–71. PMC3109883 21673879

[38] Dam T, Mahmood A, Linville K, Bailey M, Surani S. Meningeal carcinomatosis: a metastasis from gastroesophageal junction adenocarcinoma. Case Rep Med 2013; 2013: 245654. 10.1155/2013/245654. PMC3880727 24454393

[39] Mohammed A, Alzahrani A, El-Shentenawy A, Al-ezzi E. Leptomeningeal metastasis from gastroesophageal carcinoma. Forum Clin Oncol 2013; 1: 34–7.

[40] Freret M E, Boire A. The anatomic basis of leptomeningeal metastasis. J Exp Med 2024; 221: e20212121. 10.1084/jem.20212121. PMC10919154 38451255

[41] Nguyen A, Nguyen A, Dada O T et al. Leptomeningeal metastasis: a review of the pathophysiology, diagnostic methodology, and therapeutic landscape. Curr Oncol 2023; 30: 5906–31. 10.3390/curroncol30060442. PMC10297027 37366925

[42] Remsik J, Boire A. The path to leptomeningeal metastasis. Nat Rev Cancer 2024; 24: 448–60. 10.1038/s41568-024-00700-y. PMC11404355 38871881

[43] Pan Z, Yang G, He H et al. Leptomeningeal metastasis from solid tumors: clinical features and its diagnostic implication. Sci Rep 2018; 8: 10445. 10.1038/s41598-018-28662-w. PMC6041294 29992998

[44] Apostolidis L, Schrader J, Jann H, Rinke A, Krug S. Leptomeningeal carcinomatosis: a clinical dilemma in neuroendocrine neoplasms. Biology (Basel) 2021; 10: 277. 10.3390/biology10040277. PMC8066280 33800581

[45] Leal T, Chang J E, Mehta M, Robins H I. Leptomeningeal metastasis: challenges in diagnosis and treatment. Curr Cancer Ther Rev 2011; 7: 319–27. 10.2174/157339411797642597. PMC3523178 23251128

[46] Clarke J L, Perez H R, Jacks L M, Panageas K S, Deangelis L M. Leptomeningeal metastases in the MRI era. Neurology 2010; 74: 1449–54. 10.1212/WNL.0b013e3181dc1a69. PMC2871005 20439847

[47] Waki F, Ando M, Takashima A et al. Prognostic factors and clinical outcomes in patients with leptomeningeal metastasis from solid tumors. J Neurooncol 2009; 93: 205–12. 10.1007/s11060-008-9758-3. 19043775

[48] El Shafie R A, Böhm K, Weber D et al. Outcome and prognostic factors following palliative craniospinal irradiation for leptomeningeal carcinomatosis. Cancer Manag Res 2019; 11: 789–801. 10.2147/CMAR.S182154. PMC6340499 30697071

[49] Kim H, Lee E M. A retrospective analysis of the clinical outcomes of leptomeningeal metastasis in patients with solid tumors. Brain Tumor Res Treat 2018; 6: 54–9. 10.14791/btrt.2018.6.e12. PMC6212684 30381917

[50] National Comprehensive Cancer Network . NCCN Clinical Practice Guidelines in Oncology: Central Nervous System Cancers. Version 2.2026. Plymouth Meeting, PA: NCCN, 2026; https://www.nccn.org/guidelines/guidelines-detail?category=1&id=1425.

[51] Megid T B C, Baskurt Z, Ma L X et al. Leptomeningeal carcinomatosis and brain metastases in gastroesophageal carcinoma: a real-world analysis of clinical and pathologic characteristics and outcomes. J Neurooncol 2024; 167: 111–22. 10.1007/s11060-024-04576-8. PMC10978709 38372902

